# A study of vocal nonlinearities in humpback whale songs: from production mechanisms to acoustic analysis

**DOI:** 10.1038/srep31660

**Published:** 2016-10-10

**Authors:** Dorian Cazau, Olivier Adam, Thierry Aubin, Jeffrey T. Laitman, Joy S. Reidenberg

**Affiliations:** 1 ENSTA Bretagne, Lab-STICC (UMR CNRS 6285), 2 rue François Verny, 29806 Brest Cedex 09, France; 2 Sorbonne Universités, UPMC Univ Paris 06, UMR 7190, Institut Jean Le Rond d’Alembert, F-75005, Paris, France; 3 CNRS, UMR 7190, Institut Jean Le Rond d’Alembert, F-75005, Paris, France; 4 Institut des Neurosciences Paris-Saclay, CNRS UMR 9197, Université Paris Sud, Bat 446, Orsay, France; 5 Center for Anatomy and Functional Morphology, Icahn School of Medicine at Mount Sinai, New York, USA

## Abstract

Although mammalian vocalizations are predominantly harmonically structured, they can exhibit an acoustic complexity with nonlinear vocal sounds, including deterministic chaos and frequency jumps. Such sounds are normative events in mammalian vocalizations, and can be directly traceable to the nonlinear nature of vocal-fold dynamics underlying typical mammalian sound production. In this study, we give qualitative descriptions and quantitative analyses of nonlinearities in the song repertoire of humpback whales from the Ste Marie channel (Madagascar) to provide more insight into the potential communication functions and underlying production mechanisms of these features. A low-dimensional biomechanical modeling of the whale’s U-fold (vocal folds homolog) is used to relate specific vocal mechanisms to nonlinear vocal features. Recordings of living humpback whales were searched for occurrences of vocal nonlinearities (instabilities). Temporal distributions of nonlinearities were assessed within sound units, and between different songs. The anatomical production sources of vocal nonlinearities and the communication context of their occurrences in recordings are discussed. Our results show that vocal nonlinearities may be a communication strategy that conveys information about the whale’s body size and physical fitness, and thus may be an important component of humpback whale songs.

Vocal repertoires of humpback whales (*Megaptera novaeangliae*) range widely, compared with other mysticete whales, with sounds varying in duration, intensity and bandwidth. Scientists have particularly been interested in the complex stereotyped songs that males emit during the winter-spring breeding season. In these songs, the occurrence of vocal nonlinearities (i.e. instabilities) such as frequency jumps and deterministic chaos can be observed. Vocal nonlinearities can be generated by nonlinear processes involving coupled oscillators such as the paired laryngeal vocal folds of mammals (reviewed by Fitch *et al*.^1^), but it can only occur under certain conditions (e.g., high lung pressure^2^). This study will use the nomenclature defined by Wilden *et al*.^3^ to describe the following features of vocal nonlinearities. A frequency jump is an unpredictable break in the fundamental frequency trajectory that results from instability of vocal fold oscillations. The vibration rate increases or decreases abruptly, and can vary extensively in amplitude. Deterministic chaos, so named because of the erratic and random appearance of the sounds, is characterized by broadband noise-like segments that appear via abrupt transitions. However, chaos has some residual periodic energy, which appears as banding in the spectrogram, and allows it to be readily distinguished from turbulent noise.

Since the pioneering work by Payne and McVay^4^ towards the understanding of humpback whale songs, no major efforts have been made to analyze their nonlinear vocal features. Payne and McVay’s^4^ did not include any of them in their original concept of a sound unit, and these particular features continue to be omitted in major studies on classification of humpback whale sounds (see review in Cholewiak *et al*.^5^), and even from the concept of sound unit. To our knowledge, only three studies dealt with vocal-non linearities in mysticete vocal display. Tyson *et al*.^6^ reported the occurrence of subharmonics, deterministic chaos, biphonation, and frequency jumps in North Atlantic right whales (*Eubalaena glacialis*). Tervo *et al*.^7^ reported the occurrences of biphonation in the bowhead whale vocal repertoire. Mercado *et al*.^8^ performed a frequency-jump based formant analysis on humpback whale songs. As a consequence, while the presence of nonlinear phenomena in mysticeti vocal sounds has been observed and quantified, the question of their recognition as essential vocal features of humpback whale songs remains. This tendency to disregard nonlinear features may be explained by their transitory characteristics and intermittent occurrences within various sound units, which make them unreliable cues for the definition of characteristic sound units. Also, prior misunderstanding of humpback whale vocal mechanisms has hampered investigating nonlinear sound generation. Reidenberg and Laitman^9^ discovered the presence of a vocal fold homolog, overturning the long-held and widespread misconception that mysticetes lack vocal folds^10^. This misconception may have colored the classification of sound units (e.g., Au *et al*.^11^), as no distinctions have ever been made between noise-like and chaotic vocalizations, although their production mechanisms are completely distinct with respectively no vibrations and irregular vibrations of vocal folds.

The communicative function of vocal nonlinearities has been debated, but their prevalence in many mammalian vocal repertoire suggests that they could carry information^1^,^3^. Most often, vocal nonlinearities are interpreted as cues of various physiological properties of the emitter. Riede *et al*.^12^ proposed the function of an acoustic indicator for determining general physical condition in common chimpanzee (*Pan troglodytes*) communication. Fitch *et al*.^1^ and Schneider *et al*.^13^ showed how these sounds could aid in communicating animal size and individual or species identification. Mann *et al*.^14^ suggested that the manatee (*Trichechus spp*.) vocal nonlinearities could provide cues for individual recognition. Tembrock *et al*.^15^ ascribed different functions to barks of canids that depend on the ratio of harmonic to non-harmonic energy (a ratio which can be seen as a measure of chaos in vocalizations). In addition to revealing physiological status, vocal non linearities have been hypothesized to show urgency, uniqueness and virtuosity^1^. Although much has been gained in determining the mechanical production and functional context of the nonlinear sounds, it is still unknown whether their production is intentionally generated or results from uncontrolled factors. For example, intentional vocal nonlinearities could advertise greater power, superior fitness or unique skill, whereas accidental production could indicate malfunction (paralysis, injury), abnormality (asymmetrical folds), fatigue, or lack of skill/control.

## Contributing work

Recent studies^8^,^16^,^17^,^18^ have applied a production-based approach to investigate humpback whale vocal behavior. These studies aim to make the link between sound-producing anatomy and recorded vocalizations. This approach most often combines three distinct fields of investigation, i.e. functional anatomy, biomechanical modeling and bioacoustics, and has been widely applied to different animal species (e.g., doves^19^, dolphins^20^). In this paper, we adopt a similar approach to study two vocal nonlinearities (deterministic chaos and frequency jumps) in humpback whale songs from the Ste Marie channel (Madagascar). For the anatomical part of our study, the sound producing anatomy of mysticetes, including humpback whales, has been previously published^9^,^17^,^18^,^21^. The mysticete U-fold (homologous to laryngeal vocal folds) was determined to be the sound source of the fundamental frequencies. Biomechanical modeling of a vocal production system evaluates the contributions of different system parameters (e.g. pitch, formants, lung pressure or the varying interaction with tissue structures) in the overall acoustic production. Previous studies on humpback whale vocal production^17^,^18^ focused on the supraglottal part (i.e. from the vocal fold to the nose) of the respiratory tract, using a non-interactive linear source-filter model to investigate occurrences of formant-like acoustic resonances. Although this linear approximation is valid in most cases of human phonation^22^, it is a drastic simplification for animal vocal production in general. Noticeably, nonlinearities in vocal production have been proven to originate either from the vocal folds themselves (source), or from an interactive coupling between the folds and the surrounding airspaces (filter) (see Titze^23^ for details of the “source-filter” theory). In this paper, the interactive nonlinear source-filter model^23^ is used to determine potential mechanisms that generate chaos and frequency jumps. This model includes a two-mass mechanical system to simulate the physics of the paired vocal folds (the two “arms” of the U-fold), that represents a good compromise between the most basic mechanism of self-oscillating flow-induced tissue oscillation (i.e., the phase difference between upper and lower parts of vocal folds), and the more extreme mechanism involving regulation by muscle activity affecting exact flow rates and vocal fold oscillation characteristics. This vocal fold model has been connected to the vocal tracts modelled by the time-domain wave-reflection method^24^,^25^,^26^. With this model, we have simulated the transitions (also called bifurcations in literature) that occur between different vibratory regimes when system parameters (e.g. the length of vocal fold) are slowly varied. A comprehensive visualization of these transitions can be achieved by bifurcation diagrams that display different dynamical behavior depending on two varying system parameters^2^.

Validation of our production model is based on a comparison of the nonlinear structure of the simulated sounds with that of the real acoustic data. In particular, the theory of nonlinear dynamics provides the appropriate framework for understanding general voice instabilities^2^. Nonlinear dynamic analyses go beyond traditional spectral analyses and are used to calculate metrics that describe the complexity of the underlying system (for a review see Kantz *et al*.^27^). Classical tools of nonlinear methods include correlation dimension, entropy, and Lyapunov exponents^28^. Although still relatively new and unfamiliar to many researchers interested in animal vocal behavior, nonlinearities can now routinely be identified and quantified as commonplace components of numerous animal vocal communications^1^. These nonlinear metrics may be important for the study of humpback whale communication in that they can be used to characterize complex signals and determine whether differences between two signals carry important information to the receiver^29^,^30^.

## Results

### Numerical simuations

**Figure 1:**
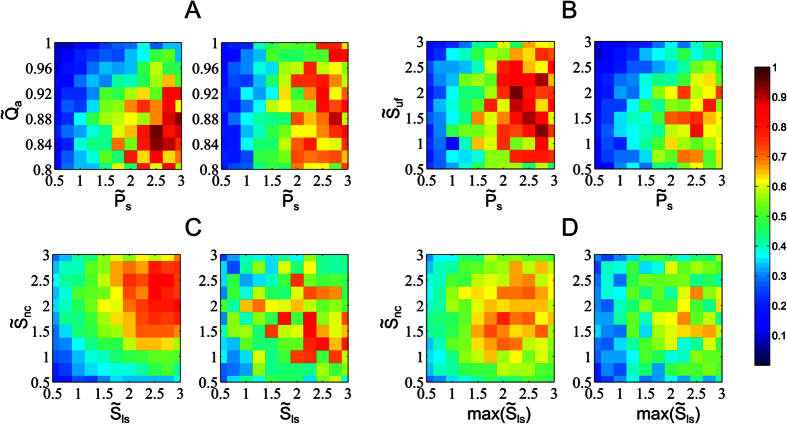
Distributions of nonlinear measures *ϕ*_*fj*_ and *ϕ*_*ch*_, respectively on the left and on the right, in four different two-parameter bifurcation diagrams: left-right U-fold asymmetry 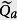/lung pressure 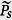 (diagram **A**), U-fold sizing 
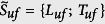
/lung pressure 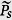 (diagram **B**), size of the nasal cavities 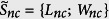/size of the laryngeal sac 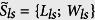 (diagrams **C,D**). The 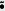 represents parameter values normalized by their nominal values from Table 3. The simulated vocalizations of diagram C have been obtained with an upward fundamental frequency modulation, and those of diagram **D** have been obtained with a laryngeal sac extension.

Results from numerical experiments are given through the two descriptors *ϕ*_*fj*_ and *ϕ*_*ch*_, measuring respectively the strength of frequency jumps and chaos in a sound unit. Their distributions in two-parameter bifurcation diagrams (Fig. 1) show different regions of vocal nonlinearities depending on the values of critical biomechanical parameters of the production model. In diagrams A and B, we focus on the subglottal pressure *P*_*s*_ (i.e. measured between the vocal fold and the carina [bifurcation of the trachea]), left-right U-fold asymmetry *Q*_*a*_, and U-fold size (i.e., *L*_*uf*_ and *T*_*uf*_). These physiological factors are varied over value ranges as shown in Table 3. Parameter values for display, labelled with a 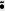, have been normalized by their nominal values from Table 3. The simulated vocalizations were then obtained by varying the normalized parameters from 1 to the values specified on the x-y axes, over the vocalization duration.

In diagram A, we can observe that as the U-fold asymmetry increases, transitions between two different dynamical behaviors of vocal fold oscillation are observed. Typically the oscillations change from a purely periodic behavior to a more complex quasiperiodic one that may contain chaos of variable strength. We also observe that both nonlinearities (i.e., frequency jumps and chaos) are reinforced when the subglottal pressure *P*_*s*_ increases. In diagram B, we see that they are also accentuated by larger oscillating fold thickness and by higher lung pressure.

Diagrams C and D of Fig. 1 focus on the characteristics of the surrounding system, specifically the lengths and widths of the nasal cavities (i.e., 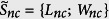) and laryngeal sac (i.e., 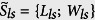). The vocalizations of diagram C simulate upward fundamental frequency modulations with the different sizes of the nasal cavities and laryngeal sac shown on the x-y axes. Modulations are obtained by increasing gradually the stiffness factor of each side of the U-fold, modeling laryngeal muscle activity that controls U-fold tension^9^. The simulated vocalizations of diagram D use a laryngeal sac extension, with a length that varies from the nominal values of *L*_*ls*_ and *W*_*ls*_ to the maximum values specified by each diagram cell, while keeping source parameters constant.

When modulating the fundamental frequency of vocal folds or the size of the laryngeal sac, different patterns of vocal nonlinearities appear (as shown in diagrams C and D of Fig. 1, respectively). The crossing between a frequency resonance of the acoustic loading and the fundamental frequency is most often the trigger for unstable oscillations of vocal folds, and added aperiodicity is observed after acoustically induced breaks. The introduction of dynamics in the source, with the stiffness-driven frequency modulation, or in the acoustic loading, with a laryngeal sac of varying size, then tends to increase the occurrence rate of fundamental frequency and formant crossings, and consequently the risk of instabilities in vocal fold oscillations. One trend that can be observed from diagrams C and D of Fig. 1, is that larger air cavities (implying more resonance frequencies) induce more and stronger nonlinearities.

### Analysis of real sounds

**Figure 2:**
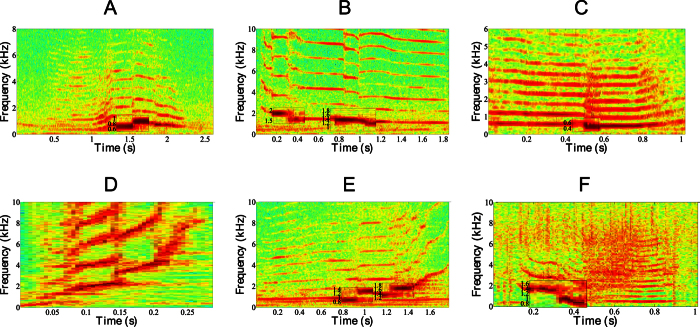
Examples of sound units with frequency jumps. The sound units (**A**–**C**,**E**,**F**) contain a box indicating the location of frequency jumps, with y-axis values. The sound unit **D** is short enough so we can distinctly visualize the frequency jump. Spectrogram parameters: sampling rate F_s_: 44.1 kHz, frame size: 22 ms (1024 samples), 50% overlap (temporal resolution: 11 ms), FFT size: 1024 samples (spectral resolution: 11 Hz), Hamming window.

**Figure 3:**
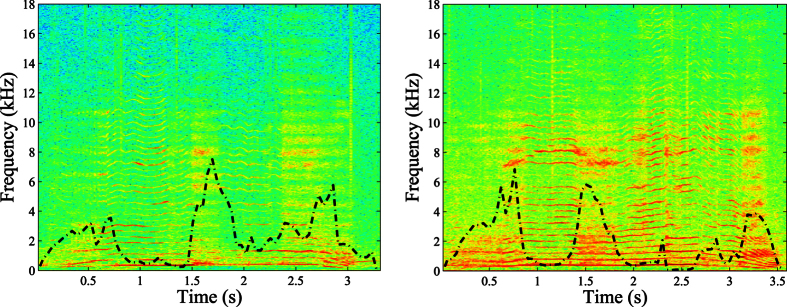
Examples of temporal distribution of chaotic segments in various nonlinear vocalizations. The solid black lines represent the *ϕ*_*ch*_ curves. Spectrogram parameters: sampling rate F_s_: 44.1 kHz, frame size: 22 ms (1024 samples), 50% overlap (temporal resolution: 11 ms), FFT size: 1024 samples (spectral resolution: 11 Hz), Hamming window.

Figure 2 represents six different examples of frequency jumps that show the diversity of occurrences. These jumps can appear in nearly flat fundamental frequency trajectories (as for sound units A, B and C) or in time varying trajectories (as for sound units D, E and F). The structural aspect of frequency jumps is highlighted, as they clearly segment each sound unit into differentiated pieces. Figure 3 represents two examples of chaotic sound units, with the *ϕ*_*ch*_ descriptor curve superimposed on them. Residual spectral components related to the previous harmonic components, or subharmonic windows, recurrently appear within chaos.

Table 1 gives results of percentage occurrences of nonlinear phenomena in each song, that are on average 35% and 41% over all detected vocalizations, respectively for frequency jumps and chaos. Deterministic chaos was the most frequently occurring nonlinearity found in our humpback whale songs, comprising 80% of all detected vocal nonlinearities, while 49% of the vocal nonlinearities were frequency jumps. In Table 1, we can also see the percentage distribution of frequency jumps and chaos over song units. We can see that chaos occur most frequently at the initiation of the signal (on average 49%), but it could also be found at the end of the signal or in multiple locations throughout the signal. Frequency jumps appear to be present more often in the middle third of sound units.

**Figure 4:**
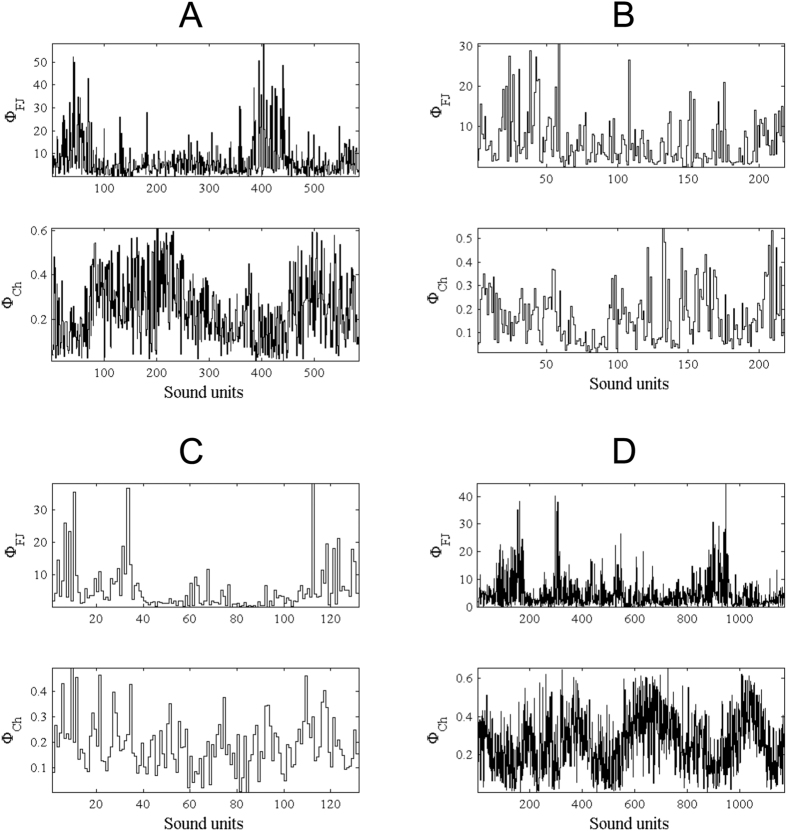
Temporal evolution of the descriptors *ϕ*_*fj*_ and *ϕ*_*ch*_ over four different songs (**A–D)** (see Table 2 for details). The difference in linewidths between the graphs is explained by their different number of sound units, from 1223 (in song **A**) to 116 (in song **B**).

Temporal evolution of the descriptors *ϕ*_*fj*_ and *ϕ*_*ch*_ over four different songs are shown in Fig. 4. All values per sound unit have been linked together to get relative sound-to-sound variations. We can observe that the descriptor values do not seem to be randomly distributed over time, although they do not follow common temporal patterns from one song to another. They are rather quite individual-specific, with clear regions of increased percentage occurrences of vocal nonlinearities.

## Discussion

Until recently, vocal nonlinearities have been disregarded as an integral vocal feature of humpback whale songs, and have never been integrated in any classification of sound units^4^,^31^. Frequency jumps and chaos have already been studied in the vocalizations of several species, including common chimpanzees (*Pan troglodytes*)^19^, manatees (*Trichechus spp*.)^14^, North Atlantic right whales^6^, and humpback whale songs^8^. For example, it was reported that 5% of manatee vocalizations contained frequency jumps and 58% contained chaos^14^, and that 19% of right whale total vocalizations contained frequency jumps and 50% contained chaos^6^. In the present study, comparable rates were found in vocalizations of four humpback whale songs from the Sainte-Marie channel (Madagascar), with percentage occurrences of 35% and 41% over all detected vocalizations, respectively for frequency jumps and chaos. In comparison to the other marine mammal vocal repertoires that have been studied, a higher proportion of frequency jumps has been observed in our humpback whale songs. This could be due to the inherent complexity of these songs (with many fundamental frequency modulations that increased frequency crossings with formants, and the occurrence of frequency jumps both in modulated and constant fundamental frequencies).

Neither chaos or frequency jumps appeared to dominate any of the vocalizations analyzed. Thus, it is possible that these features, while not prevalent, may in fact play some communicative role. This hypothesis is also supported by the non-random temporal distribution of nonlinearities over the duration of a song (see Fig. 4). Our current study goes further in this direction by providing simulation-based evidence on the vocal mechanisms originating these vocal features. Their potential communicative role(s) can then be explained by allowing their presence and variability to be ascribed differentially to anatomical and motivational factors^1^,^31^. In the following, we discuss both of these factors.

### Anatomical factors

Vocal production of all mysticeti originates in their larynx^8^. The interactive source-filter coupling model used in this article, that proved to be in good agreement with the diversity of the vocal nonlinearities observed in real vocalizations, forms the basis discussion of likely vocal mechanisms of chaos and frequency jumps. First, a high driving lung pressure tends to favor the occurrences of nonlinear processes, especially chaos, that fits well the rough sound quality with high amplitudes of chaotic vocalizations. In bifurcation diagrams A and B of Fig. 1, we found that when the subglottal pressure continues to increase and surpasses a certain threshold (referred to as the phonation instability pressure in speech production, e.g. Jiang *et al*.^32^), vocal fold vibrations repeatedly transited to chaos.

We observed that the dynamic variations of chaos within vocalizations, particularly during an “onset phase”, often reveals strong chaotic activity. This phenomenon is confirmed by other published observations, that when the subglottal pressure is close to the onset value of sustained oscillation, the lower subglottal pressure brings the vocal fold system near to its bifurcation point, creating a significant chaos-noise effect^33^,^34^. As a result, it can be expected that subglottal pressure is an important parameter that influences chaotic components of whale vocal production systems. Furthermore, during fast inflation of the laryngeal sac (i.e. high subglottal pressure), the U-shaped vocal fold of humpback whales resembles imbalanced vocal folds (i.e. left-right asymmetry between the two opposing folds), that exhibit incomplete closure during the vibration cycle^25^, resulting in a source of irregular, noisy vocalizations.

Two main types of frequency jumps were observed from simulations and recordings. Type 1 is an abrupt transition taking the form of an ascending or descending jump within a harmonic sound unit modulated in frequency (sound units A, B and C on top of Fig. 2). Type 2 is an abrupt bridge-like jump within a harmonic sound unit non-modulated in frequency (sound units D, E and F on bottom of Fig. 2). Among the vocal mechanisms investigated, several produced these types of frequency jumps. Asymmetrical biomechanical properties (in stiffness) induced a frequency jump of type 1 in the gliding harmonic structure of the U-fold, that amplifies with the subglottal pressure, as described in other publications on mammal vocal production^2^. The large oscillating surface of the U-fold likely produces temporary asymmetries between the two opposing folds. We also observed that frequency jumps tend to accentuate with the size, i.e. thickness and length, of the U-fold. In our numerical experiments, we also encountered occurrences of near-formant frequency jumps, due to cross-overs between the fundamental frequency trajectory and formants from the surrounding air cavities. Furthermore, variations of the laryngeal sac volume correspondingly vary the overall formant pattern of the supraglottal system (in particular, an inflation will lower its main formant towards the fundamental frequency of the source^18^). These variations may be responsible for frequency jumps of type 2, in which the fundamental frequency has a flat trajectory, but formants are shifted up or down until crossing this trajectory. Thus, the sizing and quick volume variations of the laryngeal apparatus of humpback whales likely favors the formation of frequency jumps.

### Motivational factors

In any understanding of a sound production system, the most interesting question may be to understand whether vocal nonlinearities are a component of intentional oral expression or are an accident result from uncontrolled factors. Many vocal functions have already been attributed to nonlinearities in animal communication, some of which fit well with the mating context of humpback whale songs. For chaos, past studies^1^,^14^ hypothesized that this feature constitutes an unpredictable signal thereby making them harder to habituate to and ignore. This, in turn, increases auditory impact on listeners by providing cues of the caller’s fitness, attractiveness, and overall health, and may assist in communicating individual identification, animal size, and urgency. In particular, the characterization of the vocal signal as a chaotic time series can give important information on the health of the animal, since the oscillation modes are related to the status of the throat tissues and to the strength of the animal. We indeed saw that chaos in vocalizations was correlated to subglottal pressure and amplitudes, which can be cues for size and fitness of the whale, and related to the muscle (e.g., intercostal and diaphragm) power and strength. Furthermore, tissues shapes of vocal apparatus are generally individual-specific in mammals, and so chaotic oscillations of these tissues could be used as an acoustic signature of whale individuals. This type of communication function may be particularly useful for humpback whale males during the mating period. Furthermore, the perception of vocal power appears to be enhanced by aperiodic vocal fold vibration, which produces a rich spectrum of inharmonic frequencies^35^. This specificity also supports the idea that chaos may function in dominance-related signalling as an index of the vocalizer body condition^36^, and as a correlate of greater vocal effort and direct auditory impact. Indeed, a higher energy cost is required for production of vocal nonlinearities. In particular it has been shown that the effort required to raise phonation intensity increases with tension imbalance^37^, and from our own results (see diagram A in Fig. 1), we saw that increasing lung pressure increases the occurrence of vocal nonlinearities.

Regarding frequency jumps, similar vocal functions can be suggested, given that this characteristic is easily distinguished from other more common vocalizations^19^. The two most common vocal features of animal communication that extract physiological information about the emitter are the fundamental frequency (source parameter) and formants (filter parameter). For example, in whales, it has been suggested that blue whales emitting a low fundamental frequency with a high amplitude were favored in mate selection by females or in competition with other males^38^, making the fundamental frequency an honest indicator of large body size. For most animal species, formant frequencies are generally considered a more reliable cue over all size, as it relates to the vocal tract length that is correlated to body size^39^,^40^,^41^. For humpback whales, we saw in our numerical experiments that the occurrence rate and strength of frequency jumps increase with vocal fold dimensions. We also reported that frequency-jumps could be induced by crossing between the fundamental frequency and formants, and thus emphasize these formants in the spectrum. Therefore, frequency jumps in humpback whale vocalizations may also serve as measurable cues of size^17^,^18^. Further to that, we can speculate that near-formant frequency jumps might be used as a dishonest acoustic cue, allowing whales to exaggerate their apparent size through remarkable inflation of the laryngeal sac, as has been observed in other species with a laryngeal air sac^42^.Eventually, if strong inter-individual differences are present in the morphology and size of humpback whale larynx, especially in the U-fold and the laryngeal sac, frequency jumps could be potentially an important cue for recognition of individual whales. Especially, unlike formants, frequency jumps (as well as chaos) are much less sensitive to distortion from underwater acoustic propagation, and thus should constitute more robust acoustic cues for individual recognition.

Our results also show that the size variations of the laryngeal sac also increase the complexity of vocalizations through the presence of stronger nonlinearities. De Boer^43^ proposed that the loss of this air sac was crucial for the development of human language, in particular because it allowed humans to articulate more complex sounds while keeping a finer control on their production mechanisms. This evolution characteristic has resulted in the disappearance of vocal nonlinearities from human language, but this study supports the expectation that humpback whales may have adapted their vocal repertoire to the constraint of their sound-producing anatomy by integrating vocal nonlinearities as an integral communication feature.

Alternatively, humpback whale vocal nonlinearities may be unintentional by-products of poor vocal technique. Frequency jumps might be a by-pass mechanical product within sound units modulated in frequency, a phenomenon known as register transition^44^. Classical singers develop technical skills precisely to smooth out these frequency jumps, as they are judged inappropriate in the aesthetics of music. More investigations, especially comparing vocal repertoires of adults and calves, will be needed to know whether a learning process allows whales to develop similar skills of controlling these jumps. It seems likely that chaos is a by-product of the sound production system mechanically transitioning from a stationary to an active state. Pathological causes could also generate nonlinearities. Diseased tissues may be unable to properly control vocal fold oscillations. Indeed, investigations of chaotic activities in human vocal production systems suggest that changes in nonlinear dynamic measures may indicate states of patho-physiological dysfunction (e.g., a noisy and breathy voice quality can result from the tension imbalance between the left and right vocal folds, diagnosed as unilateral recurrent laryngeal nerve paralysis).

Further analysis of additional instances of vocal nonlinearities on a large time-scale (e.g., as represented in Fig. 4), would help answer this question of intentionality. A systematic or random presence of vocal nonlinearities in sound units and songs would reveal a lack of physiological control in their production, and/or of motivational reasons to contextually produce them. To date, our song corpus possesses a more structured distribution of nonlinearities, indicating they appear to be intentionally produced under specific circumstances.

## Material and Methods

### Sound database

Since 2007, we have been recording humpback whales songs in the Sainte-Marie island channel, North East Madagascar, during the month of August. Recordings were done from the boat (motor off) using a COLMAR Italia GP280 hydrophone and digitalized by the Tascam HD-P2 recorder at a frequency sample (F_s_) of 44.1 kHz and 16 bits. The hydrophone was at 20 m depth, around the middle of the water column, and was taken as an average depth estimation of the singing whale position. Recordings were done close to the singers (closer than 200 m) to improve signal-to-noise ratio. Great care was taken to record only the singing focal animal, in order to prevent any confounding effect of background noise and overlapping vocalizations. In this paper, our sound database includes four songs from different whale singers. To ensure uniqueness of these singers, these songs were selected from different years, during the 2008 to 2012 period. Table 2 provides the durations (D in minutes), the number of sound units (N), the Average number of sound units per minutes (d) and the Signal-to-Noise Ratio (SNR) for each song. Measurement of SNR was done following procedure described in Mellinger *et al*.^45^, [Section 4]. It is also assumed that our database was recorded in a known and unique behavioral context that was identified as the mating song emitted during breeding season.

All sound units extracted were then screened for the occurrence of extraneous sounds through a visual inspection of the spectrogram outcomes under Adobe Audition software. All suspect features (e.g., spray whale splashes, boat noise, features from non-vocal social sounds) affecting sound units were removed. At this stage, no additional analysis was done on the nature of the vocalizations.

### Biomechanical modeling methods

**Figure 5:**
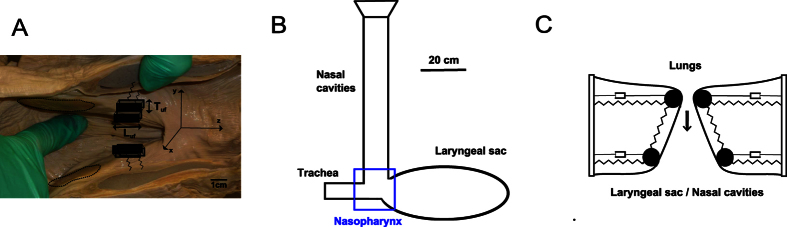
Description of the U-fold modeling. (**A**) Photo of the U-fold, with a two-mass system drawn it. (**B**) Area function of sound production system of humpback whales. This area function is not anatomically accurate, as air flow makes a U-turn above the trachea towards the laryngeal sac (see Reidenberg and Laitman^9^ and Cazau *et al*.^17^ for details). However, this turn is supposed not to play a role in our sound production model. (**C**) Two-mass model scheme. On each of these subfigures, the layngeal region is framed in blue.

A low-dimensional two-mass model was used for the U-fold, in an interactive coupling with surrounding air-filled structures. This vocal fold model can be conveniently connected to the vocal tracts modelled by the time-domain wave-reflection method^24^,^25^,^26^. With this wave-reflection method, we approximated the resonators with uniform tubes characterized by their length and area. This simplification gives direct insight into how resonance frequencies given by the tube lengths affect the location of acoustic resonances. With the low-dimensional two-mass model^2^,^46^, we divide the vocal folds tissue into upper (dorsal) and lower (ventral) portions of masses, coupled by springs (see Fig. 5 for illustration). This model captures most of the expected movement that would generate the waveforms of glottal pulses and the phonation onset, and focuses on essential features of the dynamics of vocal fold self-oscillations (i.e., the phase difference between the dorsal and ventral parts).

We now give full details on our modeling of the humpback whale vocal production system, including subglottal and supraglottal regions and the U-fold. Figure 5 presents the nominal area function used in the wave-reflection method, and the location of the U-fold within this system. The laryngeal sac has an ellipsoidal shape parametrized with the following equation, 
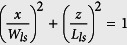
, using the coordinate axis shown in Fig. 5, and setting the default numerical parameters: *L*_*ls*_ = 60 cm (length) and *W*_*ls*_ = 30 cm (width) (Table 3). The length and cross sectional area of the nasal cavities are respectively set to *L*_*nc*_ = 80 cm and *W*_*nc*_ = 10 cm. For the two-mass vocal fold model, the physical parameter inputs are listed in Table 3. Currently, only scarce quantitative data have been published on humpback whale sound producing anatomy^10^,^47^,^48^. Nominal values for the vibrating area of the U-fold (thickness and length) can be estimated qualitatively based on anatomical analysis. All other input parameters of the vocal fold model have been drawn from literature^25^, and were adjusted to fit a nominal fundamental frequency of 120 Hz. As for models of human voice, nominal values of lumped-parameters are chosen to fit an average fundamental frequency representative of a “normal case of vocalization”. Considering the important range of fundamental frequencies covered by humpback whale sound production^8^, extensive variations around such a nominal values have been hypothesized, as stated in Table 3. Also, the laryngeal muscles around the U-fold are assumed to play a role in sound production, similar to those of terrestrial mammals, i.e. modulating the fundamental frequency by varying thickness and tension of the U-fold (see Reidenberg and Laitman^9^ for details on this hypothesis). It is also known^49^ that a small pre-phonatory opening and low damping constants support oscillations. As we also currently have no reliable values for these constants, they were reduced to remove the aphonic regimes. Eventually, the variation range set around nominal values of each of these parameters is representative of both the inter-individual variations, and the possible intrinsic variability of humpback whale vocal folds between two different vocalizations.

Furthermore, in our numerical simulations, a left-right asymmetry between the two opposing folds was also tested as a potential cause for vocal nonlinearities. Currently, anatomical evidence has not been shown to support our hypothesis for humpback whales and thus it has yet to be put to an empirical test. Nevertheless, we believe that there is considerable support for our approach from the literature. For example, studies in a range of mammals have identified occurrences of asymmetry as an explanation for nonlinearities in acoustic communication (e.g., Fitch *et al*.^1^), including many cases of voice disorders (e.g., Herzel *et al*.^2^). Furthermore, other animals that share an inflatable laryngeal sac similar to that of humpback whales, such as reindeer (Rangifer tarandus), have shown asymmetry of the sac’s position extending either to the left or to the right side of the neck^41^. Considering the tissue connection between the U-fold and the laryngeal sac in humpback whales, an asymmetrical expansion of the laryngeal sac may impact the symmetry of the U-fold’s biomechanical properties. In the two-mass model, this asymmetry is simply modeled with a linear coefficient that makes the mass and length of the left fold unequal relative to the right one. This coefficient is arbitrarily varied from 1 (symmetrical case) to 0.7 (strong asymmetry).

The subglottal pressure *P*_*s*_ is generally measured using a pressure sensor placed intratracheally, providing values of 1000 Pa for pigs^50^ and 2000 Pa for alligators^51^. For humans, a value of 750 Pa for *P*_*s*_ with a maximal source level of 90 dB (ref. 20 *μPa* at 1 m) are set^23^. On the basis of these numbers, and considering a maximal source level of 190 dB (ref. 4 *μPa* at 1 m) for humpback whales^31^,^52^, we set a nominal subglottal pressure *P*_*s*_ of 10000 Pa, and a maximal one of 18000 Pa. It is noteworthy that this dynamic pressure for vocalizations should add to the more static internal pressure in the overall respiratory system, which ensures an independent physiological control of its organs from the depth-dependent ambient pressure^53^.

The two-mass model constrains our frequency range of analysis to [0–3000] Hz. Also, only vocalizations in the egressive sense (i.e., lungs to the laryngeal sac) will be taken into account in the following, putting aside the assumption of a reverse steady self-oscillated glottal air current^9^,^18^ and its corresponding possible acoustic modification^54^, that is left for further studies. Eventually, the duration of the simulated signals was set to three seconds. The first (initial) second was discarded in order to eliminate any fluctuations due to the onset of vocalization. At the very beginning of the signal, lung pressure was increased from 0 to the desired value in 0.05 seconds, using a half period of a cosine, in order to prevent an abrupt transition (and the accompanying problems for numerical simulation). With the simulations carried out we could easily vary parameters in order to study the underlying mechanisms of the vocal nonlinearities.

### Acoustic analysis methods

With this acoustic analysis, the aim was to identify any occurrence of either a frequency jump or chaos in each vocalization of four humpback whale songs. Each song was first manually segmented in its different sound units. Only sound units with durations longer than 200 ms were considered for this analysis. Then, a semi-automatic harmonic detector is used to separate harmonic and noisy sound units. Harmonic sounds were searched for frequency jumps, and noisy sounds were inspected for chaos. The presence of a broadband spectrum with high density of unresolved frequencies is a necessary, even if not sufficient, condition for the occurrence of chaos. Eventually, each sound unit was also divided into three equal time intervals, and the presence of these nonlinearities was determined for each interval.

#### Harmonic detector

Discrimination between harmonic and noisy sound units involved the use of a simple harmonic detector that computes the element-wise product between the Fourier-transformed real signal and a theoretical comb filter. The frequency response of a comb filter consists of a series of regularly spaced notches, and aims to model a theoretical harmonic spectrum with a fundamental frequency *f*_0_. This detector has extremely-low values when the harmonic partials of the real spectrum coincide with the near-zero regions of the comb filter. In the absence of harmonic partials in the real spectrum, higher values were obtained. Mathematically, this detector then identified each of the 1024-samples long segment *T* as harmonic or noisy, respectively labelled *T*_*h*_ and *T*_*n*_, by computing


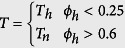


with 
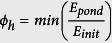
, and 
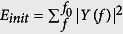
 and *E*_*pond*_ = *F*_*comb*_(*f*, *f*_0_)*E*_*init*_, where *F*_*comb*_ is a comb filter defined as 
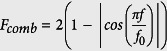
. *f*_0_ is varied with a 20-Hz step over the fundamental frequency range of humpback whales, set to [70–1200] Hz^8^. A sound unit with at least 40% of its segments that has been classified either as harmonic or noisy is itself recognized as harmonic, noisy or both.

#### Frequency jump descriptor ϕ_fj_

Frequency jumps have a recognizable property that makes them easily identifiable on a spectrogram, and our method for their detection consisted of mimicing visual identification of spectral trajectories on a narrow band spectrogram. In this work, we have used a recently developed method based on Probabilistic Latent Component Analysis^55^, and already applied to the analysis of humpback whale vocalizations^56^. This method provided us with a list of point coordinates in the spectrogram revealing the trajectories of each harmonic partial. This automatic process has been visually verified by superimposing harmonic partial estimation on the spectrogram of sound units. But the high quality of our sound database, and the smoothness of classical frequency modulations in humpback whale vocalizations made this harmonic tracking methods quite efficient. Based on these trajectories, and drawing from the spectral difference measure, we computed the relative frequency shift Δ*F*_*i*_ and the relative amplitude shift Δ*A*_*i*_ caused by a frequency jump, defined as


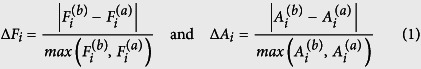


respective to the harmonic partial *i*, and where the up-scripts (*b*) and (*a*) refers to measures made before and after the jump, respectively. The descriptor ϕ_fj_ was then computed as


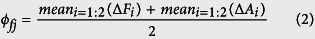


where we restricted the measure to the first two harmonics.

#### Chaos descriptor ϕ_ch_

We used nonlinear dynamics methods to identify chaos in sound units. The main difficulty in this task is that chaos can be confounded with noisy signals through spectrogram inspection, although their physical productions are completely different. Indeed, a Gaussian noise is unpredictable, whereas noise generated by a nonlinear process is predictable once the underlying nonlinear dynamics are determined. As a consequence, contrary to frequency jumps that can be easily identified from basic sound representations, chaos in signal is more difficult to identify. The reliability of our estimations was ensured by using tools already tested and validated in past studies for similar applications, as well as different individual predictions from independent descriptors. Analysis was performed with the software TISEAN^57^ that has already been used in the study of vocal irregularities in animal communication^6^,^58^,^59^. Metrics of maximal Lyapunov exponent and embedding dimension were calculated. They basically indicate whether a noisy signal is a purely random noise (maximal Lyapunov exponent of infinity and a high embedding dimension) or a chaotic signal (Lyapunov exponent between zero and infinity, and a low embedding dimension). The descriptor ϕ_ch_ was then defined by unitary normalizing each descriptor over the entire dataset, and then averaging them.

#### Thresholding

These two acoustic descriptors were applied to both simulated and recorded signals. As our goal was to evaluate absolute trends of vocal nonlinearities within these two classes of signals, as well as relative inter-class trends, we normalized these descriptors by the maximum of all descriptor values obtained in each class separately. In order to make a decision on the presence or absence of a nonlinearity in the time segments within a sound unit, we defined a threshold that when a descriptor value exceeded it, the time segment was labelled with this nonlinearity. For each class of signals (i.e., simulated or recorded), a threshold was set to 0.75.

## Conclusion

Our study gathered qualitative descriptions and quantitative analyses of nonlinearities in the song repertoire of humpback whales from the Sainte-Marie channel (Madagascar), providing more insight into the potential functions and underlying mechanisms of these phenomena. A low-dimensional biomechanical modeling of the U-fold, including a nonlinear source-filter interaction, has been used to explain the basic physics behind nonlinear production. Complementing our computer simulations, we also performed acoustic analysis to search automatically for occurrences of vocal nonlinearities through recordings of living humpback whales. Following these analyses, we have characterized nonlinearities at the sound unit and the song level, and also discussed the possibility that vocal nonlinearities could constitute an important acoustic feature in their communication framework.

Most of the research on mechanisms of vocal nonlinearities has been dedicated to human phonation, and much remains to be done for humpback whale sound production. More systematic study is necessary to further clarify the acoustic relevance of vocal nonlinearity occurrences in the mysticete communication framework. The lack of contextual observations of songs in this study, and the lack of information on individual whale vocalizations, did not allow us to address any of the proposed functional hypotheses, but the prevalence of vocal nonlinearities indicates that they are likely more than an artifact of production, and may serve communicative functions. These features are a powerful paradigm that we encourage other researchers to use. More systematic retrieval of these features through humpback whale songs should provide rich analytical support to test a behavioral communication hypothesis for the presence of vocal nonlinearities.

In future studies, collecting concurrent behavioral data would greatly increase our understanding of nonlinearities in these and perhaps other species of mysticetes, and would bring us closer to addressing many of the functional hypotheses concerning the context of their use. Further work needs to be conducted on biomechanical modeling of humpback whale U-fold to address what sounds may be originated from other regions of the fold, and whether there is sexual dimorphism of U-fold structure.

## Additional Information

**How to cite this article**: Cazau, D. *et al*. A study of vocal nonlinearities in humpback whalesongs: from production mechanisms to acoustic analysis. *Sci. Rep*. **6**, 31660; doi: 10.1038/srep31660 (2016).

## Figures and Tables

**Table 1:** Frequency of occurrence (in %) of vocal nonlinearities in each song, rounded up to the nearest percent.

	Songs	Percentage on total detected vocalizations (%)	Percentage on total detected nonlinearities (%)	Average percentage in each third of a song unit (%)
Freq. Jumps	1	33	41	21/44/35
	2	31	49	16/38/46
	3	36	57	25/35/40
	4	39	51	35/33/32
	**Mean**	**35**	**49**	**24**/**38**/**38**
Chaos	1	42	75	52/11/37
	2	34	84	47/15/38
	3	41	81	38/21/41
	4	47	79	59/8/33
	**Mean**	**41**	**80**	**49**/**14**/**37**

Note that most often a nonlinear vocalization can contain both types of vocal nonlinearities (jumps and chaos). Therefore, subcategories do not sum to give a total with only one nonlinearity. Song units (lasting more than 200 ms) were divided into thirds, and percentages calculated for each third. In the right-most column, note the average occurrence percentages are given for each third of a sound unit respectively.

**Table 2:** Details on the sound dataset: D (Song duration in minutes), N (Number of extracted sound units), *d* (Average number of sound units per minutes) and SNR (Signal-to-Noise Ratio, in dB, with mean ± standard deviation).

Song	D	N	*d*	SNR
1	41	1223	29	14.2
2	29	116	4	12.1
3	24	652	27	11.5
4	31	219	7	13.8

**Table 3:** Nominal and range of values of the lumped parameters of the biomechanical model of the U-fold.

Parameters	U-fold						Laryngeal sac		Nasal cavities	
	*L*_*uf*_ (cm)	*T*_*uf*_ (cm)	E (kPa)	*ν*	*ρ* (kg · *m*^−3^)	*η* (Pa.s)	*L*_*ls*_ (cm)	*W*_*ls*_ (cm)	*L*_*nc*_ (cm)	*W*_*nc*_ (cm)
Value	4	1	2	0.4	1020	1	50	25	80	10
Variation range	2 → 12	0.5 → 3	X	X	X	X	30 → 180	15 → 90	40 → 240	5 → 30
